# From across the Seas

**Published:** 1919-02-15

**Authors:** 


### FROM ACROSS THE SEAS.

### Visitors to Hospitals Who Abuse Privileges.

An inquiry was recently held by one of the New Zealand Hospital Boards into charges against a medical superientendent who had spoken very definitely about the conduct of some of the visitors at the hospital of which he was in charge. The charges were dismissed by the Board as being trivial. The medical superintendent, in his evidence, said: "There was no provision made for visiting at night, but a deputation from the Returned Soldiers' Department waited on me and asked could I see my way to give them a little time in the evening for those relations (fathers and mothers, sisters and brothers) who were working in the daytime and could not get along. I said, 'Certainly.' I also said if any soldier had a best girl he would like to see I would give permission. But we found that the ward was transformed into a music-hall by people who had no right to be there." The *Journal of Public Health*, in referring to this matter, causticly remarks: "It is undeniable that invalid soldiers have an indescribable fascination for a certain section of the fair sex. We have never had complaints of piano-playing and running in and out of wards occupied by, say, chronic old people, or of cases of ladies sitting for lengthy periods at the bedside of bedridden old women with whom they have had no relationship or acquaintance."

### A Hospital Shop.

A novel adjunct to the work of the Prince Alfred Hospital, Sydney, has recently been provided by the Board, in the form of a shop, at which patients and their friends and the staff may procure the small necessities which are required in their daily life, such as fruit, cakes, biscuits, sweets, tobacco and cigarettes, and items like pyjamas, slippers, brushes, and other articles of toilet which patients may not have provided. The shop has been placed in a central position in the hospital, and has proved an instantaneous success. It is largely patronised by patients, patients' friends, and the staff; and the profits, which go towards the hospital funds, are an appreciable item in the finances.

